# Enhancing Base Excision Repair of Mitochondrial DNA to Reduce Ischemic Injury Following Reperfusion

**DOI:** 10.1007/s12975-018-0680-5

**Published:** 2018-12-08

**Authors:** Roger Simon, Robert Meller, Tao Yang, Andrea Pearson, Glenn Wilson

**Affiliations:** 1 Translational Stroke Program, Neuroscience Institute, Morehouse School of Medicine, 720 Westview Dr SW, Atlanta, GA 30310 USA; 2 Exscien Corp, Mobile, AL 36688 USA

**Keywords:** Stroke, Reperfusion, Mitochondrial, DNA

## Abstract

We hypothesize that enhancing mitochondrial base excision repair (BER) capability in brain will reduce reperfusion-associated ischemic brain injury. Post-stroke reperfusion was modeled in mice via transient filament occlusion of the middle cerebral artery (60 min) (transient MCAO). Administration of a TAT-modified form of a DNA glycosylase (EndoIII) following reperfusion of the brain reduced resultant brain infarct volume. Protection was dose-dependent, BER enzyme specific, and regionally specific (more effective via the jugular vein). EndoIII is compatible with tissue plasminogen activator (tPA). The time window of a single dose of EndoIII effect is 3 h following reperfusion onset. These data suggest a novel approach to enhance protection of reperfused brain in the setting of revascularization procedures (thrombectomy or thrombolytic therapy) following stroke.

## Introduction

Drug treatments for acute stroke, based on parenchymal neuroprotection, have had remarkably few successes [1, 2]. Many such studies have been designed around the premise of inhibiting the initial phase of cell death signaling that occurs during ischemia. However, with the effectiveness of thrombolysis (tPA) and endovascular thrombectomy, vascular reperfusion is the therapeutic focus of acute stroke treatment. To optimize such reperfusion therapy, attention has now turned to investigating methods to reduce the reperfusion-induced second wave of oxidative stress in the brain as an adjunct to reperfusion therapy [3, 4]. Such an approach must, at least, be compatible with tPA. In a clinical situation, an adjunct could be pre-administered or co-administered with tPA, thereby optimizing the therapeutic time window. One approach to therapy directed against reperfusion injury is to focus on DNA glycosylases as sentinel molecules affected by reperfusion injury induced by pathological reactive oxidative species, specifically that of highly vulnerable mitochondrial DNA [5]. Cytopathologic changes, seen at the light microscopical and ultrastructural level, occur in mitochondria following ischemia/reperfusion. Such “ischemic cell change” with distention of the mitochondria is caused by mitochondrial swelling resulting from calcium loading in selectively vulnerable neurons [6] (re-altered mitochondrial morphology in CNS in the setting of ischemia, see also [7, 8]). Accordingly, our hypothesis is that reduced reperfusion-associated ischemic brain injury can be accomplished by enhancing mitochondrial DNA base excision repair capability (BER), as has been shown previously in the heart and lung (Yang, 2015) [9].

Base excision repair (BER) is the major mechanism for repairing oxidative damage to mtDNA and consists of a sequential series of enzymes responsible for removing and replacing damaged bases (uridine DNA glycosylase (OGG1), apurinic/apyrimidinic endonuclease, DNA polymerase, and DNA ligase). Mitochondrial BER activity is reduced following injurious durations of brain ischemia [10]. Furthermore, it has been shown that loss of OGG1 exacerbates ischemic injury both in vivo and in vitro [11]. In contrast, enhancing BER may be associated with neuroprotection. For example, brief non-injurious exposures to ischemia (preconditioning) result in enhanced BER activity and neuroprotection (tolerance) [10]. Indeed, genetic modulation of the first and rate-limiting step in mtDNA repair—mediated by DNA glycosylases that detect and excise oxidatively damaged purine or pyrimidine bases—coordinately regulates reactive oxygen species (ROS)-induced mtDNA damage and cell death in multiple cultured cell populations [12–17].

Therefore, as a therapy to reduce oxidative stress to neurons which occurs following reperfusion, enhancing BER activity may be a viable therapeutic approach. Accordingly, using a model of middle cerebral artery occlusion/reperfusion (MCAO) in the mouse, we offer proof-of-concept data that pharmacologic enhancement of mtDNA repair attenuates the degree of brain infarction following modeled reperfusion as an acute treatment for stroke [10]. Neuroprotection was observed in the absence and presence of tPA; accordingly, this approach has utility as an adjunct therapy for stroke.

## Materials and Methods

### Stroke Model

**Fig. 1:**
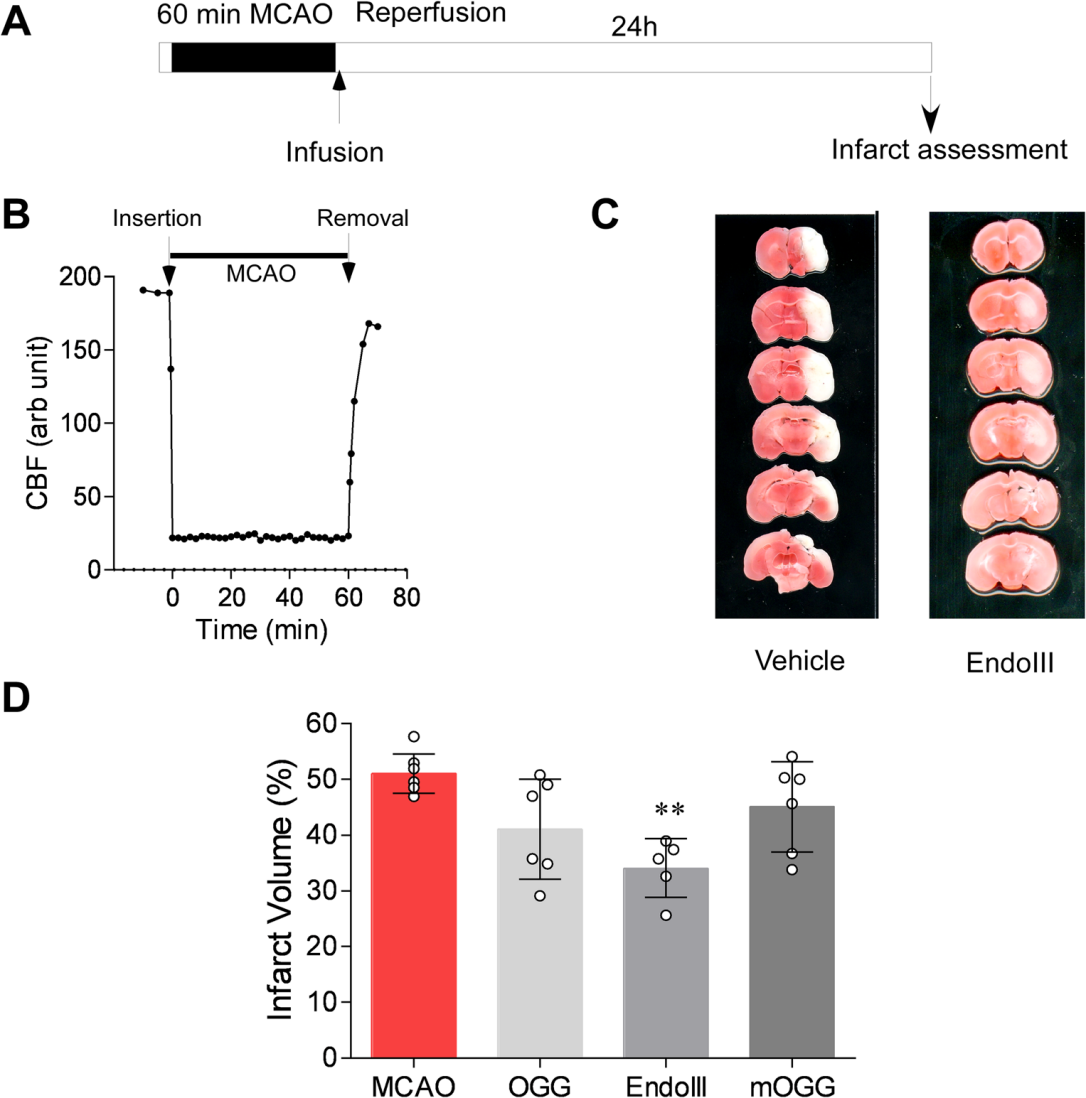
EndoIII is an effective neuroprotective agent in ischemic brain. **a** Schematic of experimental design. For experiments in Fig. 1, EndoIII or vehicle was administered immediately following removal of the MCA occluding filament. **b** Laser Doppler recording of temporal cortex blood flow following insertion of filament and withdrawal. Animals show at least 80% recovery in blood flow. **c** Exemplary TTC staining images of the brain following MCAO (60 min) and 60 min MCAO followed by EndoIII administration. **d** Drugs were administered as a single i.v. bolus at the time of reperfusion onset following 60-min middle cerebral artery (MCAO) occlusion. Infarct volume is presented as percent of hemisphere infarcted in mouse brain 24 h after treatment (% infarct volume). Animals were treated with vehicle (control) or with a single intravenous dose (100 μg/mouse) of the MT DNA glycosylase EndoIII given by tail vein. mOGG is an enzymatically inactive OGG.

**Fig. 2:**
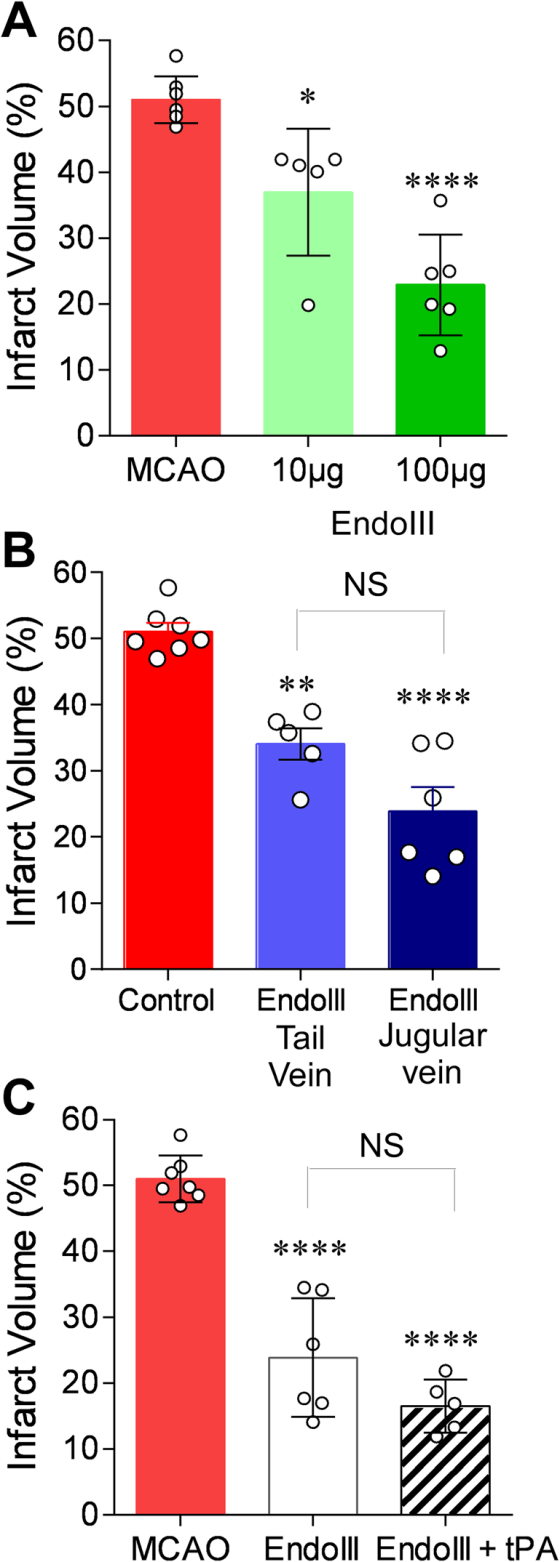
EndoIII dose response and effect as adjuvant therapy for tPA. **a** Effect of 100-μg and 10-μg dose of EndoIII on infarct volume. Animals were administered agent by jugular vein infusion immediately following filament withdrawal. **b** Effect of tail vein administration vs jugular vein administration of EndoIII. Data in the third group is repeated in **c**. Jugular vein administration was not significantly different from tail vein administration but did appear to produce more significant protection compared to harmful MCAO alone. One-way ANOVA with Tukey’s post hoc test for multiple comparisons: **p < 0.01 and ****p < 0.001 vs. vehicle control; NS: not significantly different tail vein vs jugular vein administration. **c** Administration of intravenous tPA (4.5 mg/kg) just prior to EndoIII did not reduce the protection observed following EndoIII alone. Each bar represents the mean ± SD of 5–7 animals; individual data are given as black and white circles. One-way ANOVA with Tukey’s post hoc test for multiple comparisons: *p < 0.05, **p < 0.01; ***p < 0.005, and ****p < 0.001 vs. vehicle control; NS: not significantly different vs. EndoIII

**Fig. 3:**
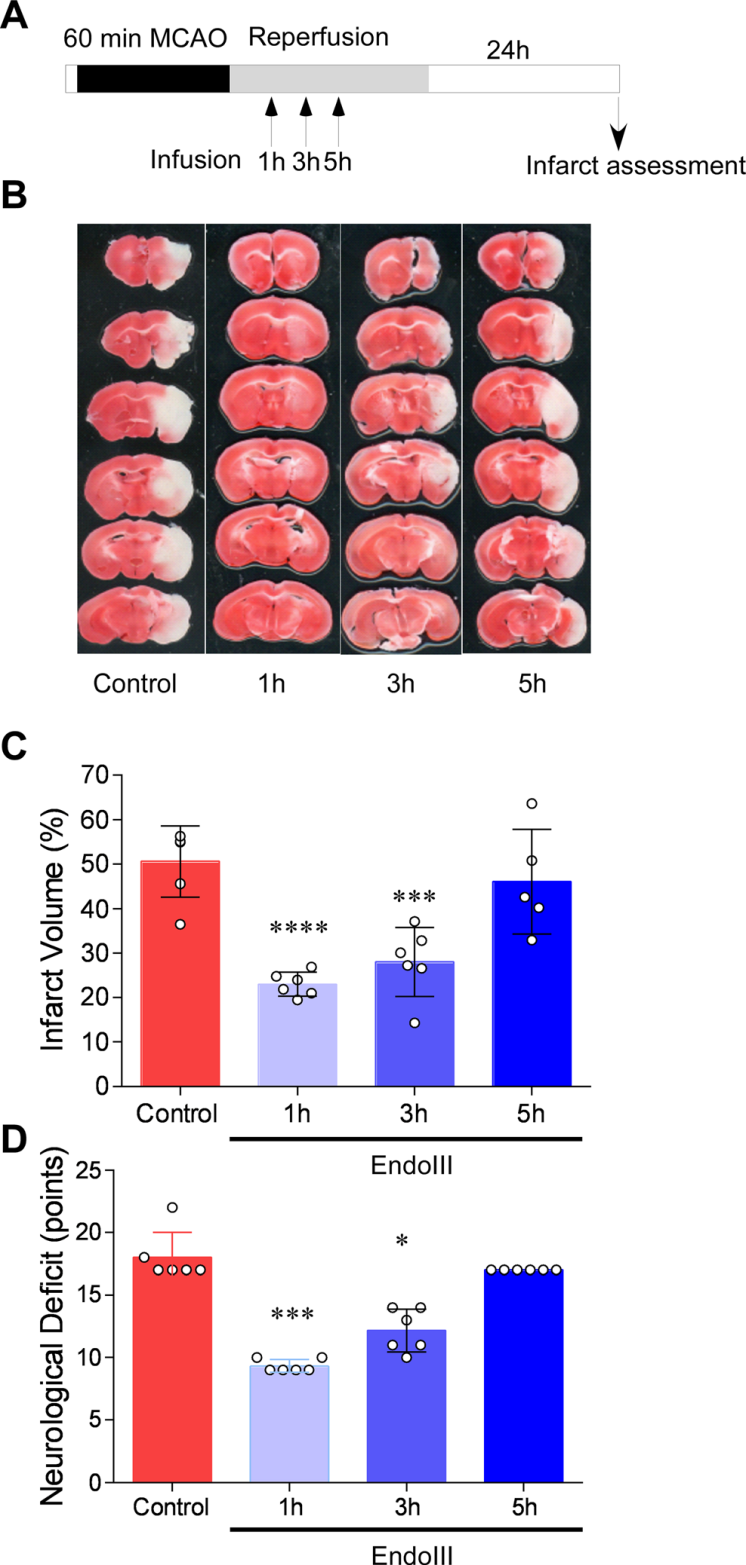
EndoIII has an effective therapeutic time window of up to 3 h in mice following transient 60-min MCAO. **a** Schematic diagram of experiments, showing administration timing of EndoIII with respect to MCAO. **b** Representative images of brain TTC staining following 60-min MCAO, following suture removal and administration of a single infusion of EndoIII 1, 3, or 5 h later. **c** Quantification of infarct volume (% non-infarcted hemisphere). Each bar represents the mean ± SD of 5–6 animals; individual data are given as black and white circles. Data were analyzed with one-way ANOVA using Tukey’s post hoc test for multiple comparisons: *** < 0.005; ****< 0.001 vs. vehicle control. **d** Assessment of motor function prior to sacrifice using a 28-point scale of focal motor assessment including body symmetry, gait, climbing, griping at 45°, circling behavior, forelimb symmetry, and whisker response [30, 29]. Controls were untreated. 1, 3, and 5 h refers to time after reperfusion at which the neuroprotective fusion protein was administered. Each bar represents the mean ± SD of 5–6 animals/time point (*** < 0.005 and * < 0.05 vs. control MCAO: Kruskal-Wallis test with post hoc Dennett’s test)

**Fig. 4:**
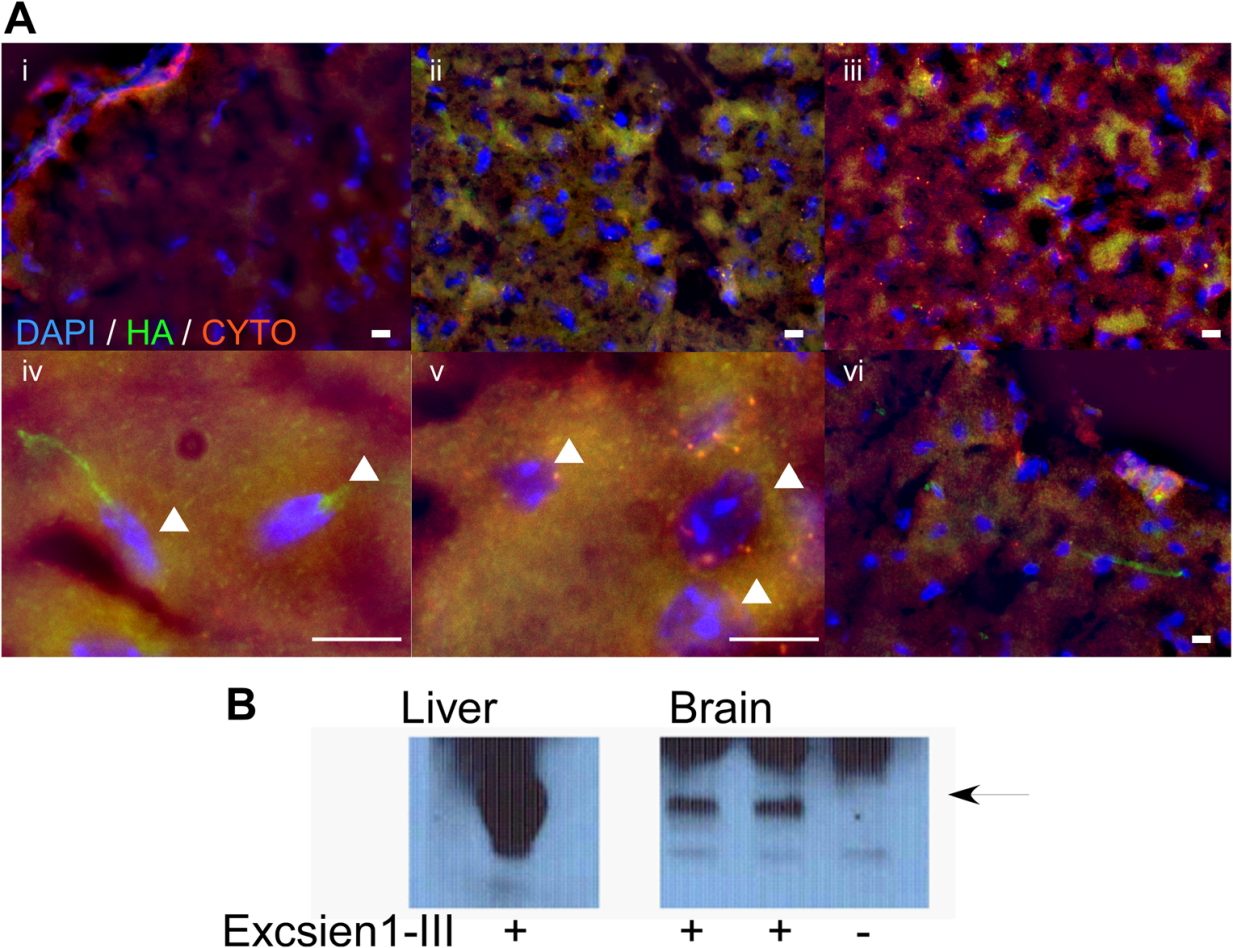
**a** Perinuclear HA staining is observed in Exscien1-III-treated animals (100 μg/intra-jugular IV: 1 h recoevery). Mito colacalization is shown with cyto C double staining v. Sections (10 μm) were processed for HA staining and cytochrome C. Images are brain 1 h following EndoIII administration following either 60-min MCAO (i–v) or control (vi). i–iii Staining of HA and cytochrome C following iv administration of EndoIII following 60-min MCAO. i contralateral cortex, ii ipsilateral cortex (penumbra), iii ipsilateral cortex (core). Images were obtained using 40× objective bar denotes 10 μm. iv 100× ipsilateral cortex (core), v 100× ipsilateral cortex (penumbra). White arrows denote cell nuclei bar = 10 μm. iv ipsilateral control following EndoIII (no MCAO). Blue DAPI, green HA tag (EndoIII), red CytoC. **b** EndoIII fusion protein or vehicle administered intravenously and detected in brain lysate (right) by Western blotting with an anti-body to the HA tag

All animal experimental protocols were reviewed and approved by the Morehouse School of Medicine Institutional Review Board (IRB), in accordance with guidelines established by the Association for Assessment and Accreditation of Laboratory Animal Care International. The manuscript was written in accordance with the Animal Research: Reporting in vivo Experiments (ARRIVE) guidelines (http://www.nc3rs.org.uk/arrive-guidelines; accessed May 2017). Experiments were first performed on male C57 mice (Charles River) obtained at 8 weeks in age and acclimatized for 1–2 weeks in the animal facility. A parallel study in female mice (synchronized by exposure to male bedding) was also performed. As we are studying reperfusion injury, stroke was modeled, in anesthetized animals (inhalation: 1.5% isoflurane, 70% N_2_O, 28.5% O_2_), using the transient middle cerebral artery occlusion technique [18] with reperfusion modeled by suture removal. With these techniques, the timing and degree of reperfusion were under direct control as was the period of ischemia. Ischemia and reperfusion were documented by transcranial Doppler as we have previously shown [19–24] (Fig. 1). As is routine, rectal (core) temperature was maintained at 37 ± 0.5 °C with a thermostatically controlled heating pad and heating lamp. Animals were randomly allocated to treatment groups. Following our initial assessment of the glycosylases (Fig. 1d), experiments were then performed blinded, whereby the surgeon did not know whether drug or vehicle was being administered (from a numbered single use tube). EndoIII, as the most effective agent, was used in subsequent experiments (Figs. 2, 3, and 4).

The fusion protein constructs (see below) were administered intravenously, in a single bolus of 10 μg or 100 μg/mouse [25], immediately following, and at 1, 3, or 5 h after removal of the suture occluding the MCAO. Parallel studies were performed in the presence of simultaneously administered tPA (4.5 mg/kg i.v.). For blinded studies, EndoIII or vehicle were aliquoted into single use tubes randomly labeled numerically.

### Volumetric Infarct Quantization

Infarct volume determination utilized vital dye (TTC; 2,3,5-triphenyltetrazoliumhydrochloride) staining [26], which does not produce data different from conventional histology (H&E) [26, 27]. Removed brains were sectioned coronally at 1 mm intervals. Sections were immersed in TTC in saline for 20 min at 37 °C and then transferred to 4% paraformaldehyde for 15 min. Six sections were analyzed for infarction size by a blinded observer using NIH-imaging software. The infarction area in each section was calculated by subtracting the normal area that stained with TTC in the ischemic hemisphere from the area of the non-ischemic hemisphere. Infarction volume was calculated by summing the infarction areas of all sections and multiplying by the slice thickness [28].

### Neurological Assessment

Mice were assessed using a 28-point motor scale, designed for mouse, including subjective and objective features, which has a correlation of 0.841 with infarct volume [29, 30]. The examiner was blinded as to the treatment.

### Chemicals

The glycosylase (endonuclease III protein) was expressed in *E. coli* strain C41 (purified by metal chelate affinity chromatography and collected by buffer exchange on a gel-filtration column). The Endo III fusion protein, containing a mitochondrial import sequence, and a protein transduction domain appended to endonuclease III, was a gift of the Exscien Corporation, Mobile, AL. Fusion proteins were coupled to a TAT sequence to facilitate cell uptake, to a hemagglutinin (HA) tag, and a polyhistidine tail for purification. MOGG is an enzymatically inactive OGG1 generated by site-directed mutagenesis with overlap extension [31].

In the context of the STAIR criteria, we assessed sample sizes for this study, based on power analysis of our previous studies in mice [20]. Group sizes were calculated for a modest effect with power alpha = 0.05 and 1-beta of 0.8. A post hoc power analysis of this study was also performed—data in Fig. 3a (51.03 ± 3.5% vs 23.99 ± 9% infarct volume as mean ± SD) has a calculated effect size of 4, alpha of 0.001, and an implied power (1-beta) of 0.97 (G3Power). All data were analyzed using Graphpad Prism v 6.0. All data are reported as mean (± standard deviation). Data were analyzed by ANOVA and post hoc tests as described in the text or figure legend. Significance was set at *p* < 0.05 (absolute *p* values are given). For non-parametric data (neurological scores), a Kruskal-Wallis test with post hoc Dunn’s test was used.

Re-inclusion and exclusion criteria: inclusion required that laser Doppler measurement showed blood flow in the cortex served by the middle cerebral artery to be reduced more than 80% following filament insertion, and that blood flow is restored upon withdrawal (Fig. 1b). Exclusion: death (there were none).

Thus, these methods adhere to current NINDS RIGOR guidelines for research including attention to Power analysis, randomization and blinding, adequate and correct statistical analysis, exclusion and inclusion criteria.

## Results

We used the 60-min transient middle cerebral artery occlusion model to mimic conditions of reperfusion following thrombectomy or clot lysis (Fig. 1). Brain infarct volume was determined to be 51.0% (3.5%) mean (± SD) 24 h following a 60-min MCAO, which is similar to our previously reported studies [20, 32] (Fig. 1c, d). Hemorrhage is easily seen in these 1-mm sections; none was seen. We first tested the protective effect of two enzymes involved in mitochondrial repair, OGG1 and EndoIII administered following filament withdrawal (Fig. 1d) administered via tail or jugular vein. Administration of EndoIII, but not OGG1 via the tail vein, caused a significant reduction in infarct volume (34.1% (5.3) *p* = 0.0010 and 41.1% (8.9) *p* = 0.0991, respectively (Dunnett’s post hoc vs MCAO only): Fig. 1d). A mutant OGG1 construct did not reduce infarct volume (45.1 (8.1) *p* = 0.5476: Fig. 1d). Since EndoIII appeared more potent, we further investigated this protein.

We investigated the effect of administration on neuroprotection by EndoIII. The higher dose of 100 μg/animal was more effective at reducing infarct volume compared to the lower dose of 10 μg/animal; however, both cause a significant reduction in infarct volume compared to untreated animals (22.9 (7.6) *p* = 0.0001, 37.0 (9.6) *p* = 0.0136, respectively (post hoc Dunnett’s test vs MCAO only): Fig. 2a). The dose of 100 μg was the maximal effective dose in myocardial ischemia as well [25]. Administration of EndoIII (100 μg/animal) was more protective when administered via jugular vein (Fig. 2b) compared with tail vein administration (Fig. 1d). The effect in female mice was studied in parallel experiments; EndoIII (100 μg/animal) administered via jugular vein was equally neuroprotective compared with males (*P* < 0.05). Finally, we tested a mutant (inactive) EndoIII, and this resulted in a significantly larger infarct volume compared to active EndoIII (11.4 ± 1.9 vs 15.7 ± 2% of infarct volume, *n* = 5 each, *P* < 0.01).

We determined whether EndoIII would be compatible with tPA administration [3]. When EndoIII was administered with tPA, there was a slightly larger reduction in infarct volume, but this was not significant to EndoIII alone (23.89 (9.0) vs 16.52 (4.05) *p* = 0.175). However, administration of tPA with EndoIII significantly reduced infarct volume compared to MCAO-treated animals (both *p* < 0.0001) (Fig. 2c). Together, these data show EndoIII to be compatible with tPA co-administration.

One significant challenge of many neuroprotective strategies has been the time window of therapeutic effects. Most patients are treated several hours following the initial stroke event. By focusing on reperfusion (i.e., when tPA is administered or endovascular clot removal performed), this limited time window is avoided; however, we determined whether delayed administration of EndoIII after reperfusion would still reduce re-perfusion injury as might occur following prolonged time to recanalization especially with thrombectomy or the time necessary for thrombolytic clot dissolution. Animals were treated 1, 3, and 5 h following reperfusion, and infarct volume was determined 24 h later (Fig. 3a). As can be seen in Fig. 3c, delaying EndoIII 1 or 3 h following reperfusion still produced a protective effect; however, the protection was lost after a 5-h delay (22.97% (2.7) *p* = 0.0001, 28.02% (7.7) *p* = 0.0005, 46 03% (11.7) *p* = 0.7804, respectively (Tukey’s multiple comparison test). Assessment with a 28-point scale of motor function assessed just prior to sacrifice shows that functional improvement correlated with infarct reduction (Fig. 3d). Specifically, neurological deficits following 60-min MCAO were reduced if EndoIII was administered 1 (*p* = 0.002) or 3 h (*p* = 0.05) following reperfusion, but not 5 h following reperfusion (*p* > 0.9999) (Kruskal-Wallis test with post hoc Dunn’s test).

To test the brain penetrance of EndoIII, we show Endo-III in a perinuclear pattern in the brain with ICC (but not when the primary antibody is omitted) (Fig. 4a). Using Western blotting, we also show EndoIII in brain lysates when EndoIII is administered. Also demonstrated but not illustrated are experiments showing subcellular fractionation showing EndoIII in brain cytosolic and mitochondria subcellular compartments; the protein integrity in the brain was confirmed as IP of the HA tag and Histo IB showing the same length as exogenous EndoIII (not shown). Together, these data suggest that EndoIII is brain penetrant and associates with the mitochondria.

## Discussion

The major finding here is that intravenous administration of a mitochondrial base excision repair enzyme attenuates infarct size and neurologic deficit in the setting of reperfusion and does so when administered up to 3 h after modeled reperfusion therapy for stroke (Fig. 3). Repetitive dosing, e.g., in the setting of tPA administration followed by late thrombectomy, as supported by the recently presented DAWN trial, might be indicated. Multiple dosing is now under study. We show that EndoIII therapy is compatible with tPA and thus EndoIII could be pre-administered or co-administered with tPA.

These results fit with our hypothesis that stroke attenuation by EndoIII is due to repair of mtDNA injury damaged by ROS. It has been shown experimentally in the heart [33] and brain [34, 35], that a burst of oxygen-free radicals occurs early in reperfusion. Attenuation of reperfusion itself can be neuroprotective. Intermittent re-occlusion of the artery that perfuses the ischemic vascular bed, a phenomenon in the heart and brain known as “postconditioning” [20, 36], attenuates end organ injury. In our experience, such postconditioning is effective if implemented within the first 30 min of reperfusion [20]. Thus, we expected at least this time window of effectiveness. The time window of effect seen here, however, is 3 h following reperfusion (Fig. 3c). Functional outcome is improved in the same time frame (Fig. 3d).

The significance of these studies lies in its focus on reperfusion injury in ischemic brain such as that occurring in the setting of acute revascularization therapy following stroke. In acute ischemic brain, reperfused by clot dissolution with tissue plasminogen activator (tPA) or mechanical clot removal by endovascular therapy, we postulate that mtDNA repair drugs, by attenuating reperfusion-induced mitochondrial injury, will potentiate the neuroprotective effect of restoring brain perfusion. Clot dissolution produces reperfusion, and successful reperfusion is associated with better functional outcome [37, 38]. Mechanical clot extraction, while yielding improved reperfusion over tPA clot dissolution, did not show increased clinical benefit over clot dissolution therapy in three US trials [37, 39]; however, a new retrieval device trialed in the Netherlands and now used in major US centers has shown clear benefit of acute clot retrieval technique [40]. With acute reperfusion of the brain by way of thrombolysis or endovascular therapy, an additional restorative approach to ischemic injured, but reperfused brain, may be beneficial [3]. The innovation displayed by the data presented here relates to the concept that drugs that enhance mtDNA repair could have therapeutic effects in acute stroke using molecules directed against a novel pharmacologic target in acute reperfused brain ischemia. A mitochondrial focus for therapy of ischemic brain injury recently encompasses upregulation of Parkinson’s disease-associated protein DJ-1 [41].

Many early studies of endogenous tPA suggested a detrimental effect on hypoxic or ischemic brain [42, 43], and later, tPA was discovered to open the blood-brain barrier [44]. Accordingly, we separately assessed the possible modulatory effect of tPA induced reperfusion upon BER neuroprotection by administering tPA just prior to EndoIII glycosylase treatment (Fig. 2b). We found that the presence of tPA does not affect base excision repair neuroprotection (Fig. 2b). Recent studies of endogenous tPA and exogenously administered tPA support a neuroprotective effect of tPA (independent of plasminogen cleavage) and dose efficacy in mouse in the same range as humans (therapeutic administration of tPA dose of 4.5 mg/kg in mouse) [45].

Delivery systems may be necessary to permit therapeutic proteins to enter the brain. The data presented here incorporating TAT fusion proteins to facilitate mt-BER uptake into ischemic brain when delivered by an intravenous route are in concert with our studies using TAT and the anti-apoptotic protein Bcl-w (TAT-Bcl-w) with an HA tag in neuronal cultures. Further, TAT systems have been used to deliver the anti-apoptotic protein Bcl-xL to brain in a transient focal ischemia/reperfusion model; attenuated injury was observed even with a 45-min interval between reperfusion onset and intraperitoneal administration of TAT-Bcl-xL [46]. Here, we show that the EndoIII TAT fusion protein is neuroprotective in that time frame (Fig. 3c).

We performed an a priori power analysis of previous 60-min MCAO data to determine group sizes. The effect size was set at moderate to ensure that a meaningful reduction in infarct volume would be detected. Of note, experiments in Figs. 1, 2, and 3 are independent replicates, confirming that the dose and administration route of EndoIII are protective in our model. A 24-h interval of assessment after the protection administration was selected as we have published data comparing infarction following 60 min of transient focal ischemia (as used here) in brains examined 24 or 96 h later; there is no difference [47]. Further, the data from Michael Chopp’s lab analyzed the temporal profile of MCA damage in H&E sections over time in rat 6 to 168 h after permanent or transient focal ischemia (tMCAO) with suture occlusion and found that “after 24 h no significant difference in the size of the cortical lesion was detected …” [48]. The 24-h lesion data are supported by behavioral data showing a reduction in neurological deficits in EndoIII-treated animals (Fig. 3d).

Of note, our proposed stroke therapeutic differs from previous approaches to develop drugs for the treatment of acute stroke. While supportive data of EndoIII efficacy has also been shown in modeled myocardial ischemia/reperfusion [25], we do not propose here a new standalone stroke therapy. Our treatment approach builds on effective proven treatment: revascularization. Emergency revascularization therapy is effective; acute stroke centers are widely established, and a rapid response to stroke symptoms is aggressively disseminated to the public. Thus, the acute treatment therapeutics and the stroke treatment population are already identified. However, while revascularization therapy is effective for acute stroke (the only treatment approved for acute stroke), maximizing reperfusion has not always been associated with a maximized brain protective effect [37, 39, 49]. Our mt-targeted DNA repair drugs are offered to add tissue protection to reperfusion therapy and address this limitation.
